# The novel ALK K1150dup mutation mediates resistance to frontline lorlatinib and retains sensitivity to gilteritinib

**DOI:** 10.1038/s41698-026-01477-z

**Published:** 2026-05-13

**Authors:** Mai Nagasaka, Francesco Facchinetti, Ludovic Bigot, Floriane Brayé, Matthew R. Groves, Juvenal Yosa, Anthonie van der Wekken, Mihaela Aldea, Benjamin Besse, David Planchard, Charles Naltet, Ryohei Katayama, Ken André Olaussen, Yohann Loriot, Luc Friboulet

**Affiliations:** 1 Gustave Roussy, Université Paris-Saclay, Inserm, Villejuif, France; 2 Division of Experimental Chemotherapy, Cancer Chemotherapy Center, Japanese Foundation for Cancer Research, Tokyo, Japan; 3 IHU PRISM National PRecISion Medecine Center in Oncology, Gustave Roussy, Villejuif, France; 4 Département de Médecine Oncologique, Gustave Roussy, Villejuif, France; 5 Protyon BV, Groningen, The Netherlands; 6 Department of Chemical and Pharmaceutical Biology, Groningen Research Institute of Pharmacy, University of Groningen, Groningen, The Netherlands; 7 Genomics for Health in Africa (GHA), Africa-Europe Cluster of Research Excellence (CoRE), Accra, Ghana; 8 Department of Pulmonology and Tuberculosis, University of Groningen, University Medical Centre Groningen, Groningen, The Netherlands; 9 Department of Respiratory Diseases, Hôpital Paris Saint-Joseph, Paris, France; 10 Department of Computational Biology and Medical Sciences, Graduate School of Frontier Sciences, the University of Tokyo, Tokyo, Japan; 11 Département d’Innovation Thérapeutique (DITEP), Gustave Roussy, Villejuif, France

**Keywords:** Cancer, Cell biology, Drug discovery, Oncology

## Abstract

Lorlatinib, a third-generation ALK tyrosine kinase inhibitor (TKI), effectively targets most single ALK mutations in ALK-positive non-small cell lung cancer (NSCLC), while acquired resistance remains a major clinical challenge. In this study, we identified a novel ALK K1150dup mutation in a patient who progressed on first-line lorlatinib. Functional studies using EML4::ALK-dependent Ba/F3 cells demonstrated that ALK K1150dup confers resistance to lorlatinib and other ALK-TKIs including NVL-655 (neladalkib), with sustained ALK phosphorylation and downstream signaling. Importantly, multi-kinase inhibitor gilteritinib potently inhibited ALK phosphorylation and suppressed proliferation of ALK K1150dup-mutant cells. These findings reveal K1150dup as a previously unidentified mechanism of resistance to first-line lorlatinib, and highlight gilteritinib as a potential therapeutic option for patients harboring this mutation. Notably, they also challenge the prevailing assumption that first-line lorlatinib precludes the emergence of single on-target ALK resistance mutations.

## Introduction

*ALK* gene alterations, including chromosomal rearrangements and point mutations, lead to aberrant ALK activation that drives downstream oncogenic signaling. ALK fusions are present in 3–5% of patients with non-small cell lung cancer (NSCLC)^1,2^. These fusions promote ligand-independent dimerization, resulting in constitutive activation of downstream signaling pathways, including RAS–MAPK and PI3K–AKT^3,4^. To date, numerous ALK fusion partners have been identified^5^. Among them, EML4::ALK is the most common fusion, accounting for ~85% of ALK-positive NSCLC cases^6,7^.

ALK-positive NSCLC depends on ALK signaling for growth and survival, making them highly sensitive to ALK tyrosine kinase inhibitors (TKIs). Currently, six ALK-TKIs have been approved by the US Food and Drug Administration (FDA) for advanced ALK-positive NSCLC, and several others are in clinical development^8^. Crizotinib, the first-generation ALK-TKI, demonstrated significant clinical efficacy compared with conventional chemotherapy, but most patients eventually develop acquired resistance within the first or second year of treatment^9,10^. This resistance is often driven by bypass pathway activation or secondary ALK mutations. Notably, crizotinib has limited potency against a broad spectrum of secondary resistance mutations, including L1196M, G1202R, and G1269A^11^. Second-generation ALK-TKIs, such as ceritinib, alectinib, brigatinib and ensartinib, were developed to overcome many crizotinib-resistant ALK mutations and have significantly improved clinical outcomes. However, almost all patients eventually develop resistance to second-generation ALK-TKIs, most frequently due to the G1202R mutation^12^. Lorlatinib, a third-generation ALK-TKI, was developed to overcome a broad spectrum of resistance mutations that limit the efficacy of second-generation ALK-TKIs. While it demonstrates potent activity against most single ALK mutations, its long-term efficacy is frequently limited by the emergence of acquired resistance, such as compound mutations (*i.e*. multiple mutations occurring on the same allele)^13,14^. Recently, NVL-655 (neladalkib) has been developed to be active against ALK compound resistance mutations and to have a better tolerability profile compared to previous drugs^15^. Of note, the FDA granted breakthrough therapy designation to neladalkib for patients who have received at least 2 prior lines of ALK-TKIs, based on the results of the 1/2 ALKOVE-1 trial (NCT05384626).

The development of serial generations of ALK-TKIs has reshaped the treatment strategies for patients with ALK-positive NSCLC, moving from sequential to “best inhibitor first” strategies^16^. The unprecedented clinical outcomes observed with lorlatinib in the CROWN trial, with a median progression-free survival (PFS) not reached after five years of follow-up, positioned the third-generation TKI as the new first-line treatment^17^. As anticipated experimentally^13^, preliminary data from the CROWN trial showed that lorlatinib given as an upfront inhibitor precluded the emergence of single and compound ALK resistance mutations^17^.

Here, we report the identification and characterization of the ALK K1150dup mutation in biopsies from a patient with ALK-positive NSCLC progressing on first-line lorlatinib. This study aims to investigate the impact of the K1150dup mutation on lorlatinib progression and to explore potential therapeutic strategies to overcome resistance.

## Results

### Clinical case

During the spring of 2018, a 56-year-old woman was diagnosed with a stage IV ALK-positive adenocarcinoma with nodal, hepatic and adrenal involvement. She had a history of smoking (12 pack years, stopped 26 years before) and eight years before she was treated with thyroidectomy and radioactive iodine therapy for a papillary thyroid cancer.

**Fig. 1:**
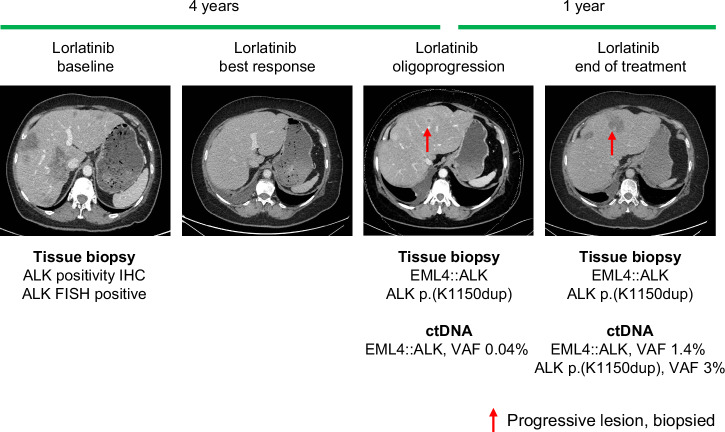
Clinical case. Clinical and molecular evolution of the patient during treatment with lorlatinib. VAF variant allele frequency, ctDNA circulating tumor DNA.

Given her ALK status, she was enrolled in the CROWN phase 3 clinical trial, evaluating first-line lorlatinib versus crizotinib^17^, and was randomized in the lorlatinib arm. Treatment with the third-generation ALK inhibitor led to a marked and prolonged disease response (RECIST -81%), lasting for four years (Fig. 1). Of note, the lorlatinib dose was progressively reduced from 100 to 75 to 50 mg daily for adverse events such as blurred vision, paresthesias, peripheral oedema. In May 2022, isolated disease progression was detected in the liver. The lesion was biopsied and treated with radiofrequency ablation. The tissue biopsy confirmed the presence of an EML4::ALK variant 3 transcript, also found in the circulating tumor DNA (ctDNA) at a variant allele frequency (VAF) of 0.04%. Of note, the tissue biopsy revealed a previously unreported ALK mutation p.(K1150dup) (c.3347_3349dup).

Given the oligoprogressive nature of the disease, lorlatinib was maintained for an additional year, when liver progression was documented in terms of multiple and large metastases. In addition to the ALK fusion, both tissue and liquid biopsies at this timepoint revealed the same ALK mutation p.(K1150dup) (VAF in ctDNA 3%). Whole-exome sequencing of the progressing tissue biopsy showed a VAF of 26% for ALK K1150dup, with no evidence of concomitant ALK amplification. This level is consistent with a subclonal event, which may represent an acquired resistance mutation emerging under treatment pressure. Considering tumor purity and potential intratumoral heterogeneity, this finding is compatible with the expansion of a resistant clone at progression. The patient experienced primary resistance to an investigational drug and then was treated with chemotherapy. She passed away nine months from lorlatinib progression, six years after the diagnosis of advanced disease.

### ALK K1150dup mutation confers resistance to lorlatinib in Ba/F3 cell models

**Fig. 2:**
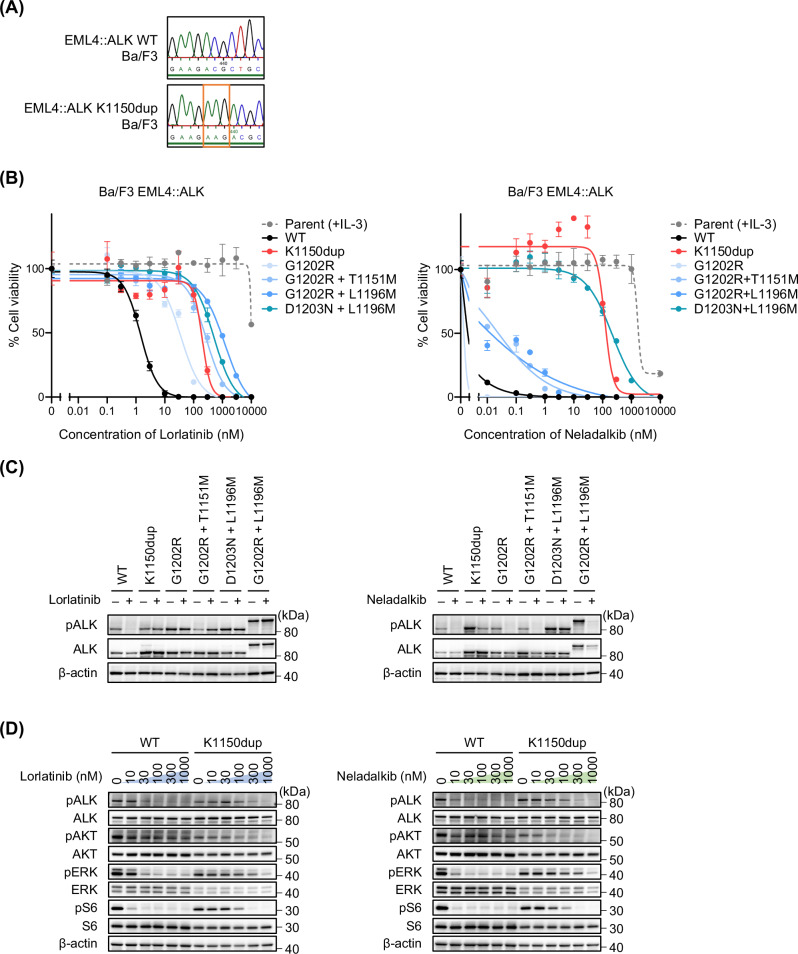
Functional analysis of the ALK K1150dup mutation in Ba/F3 cell models. **A** Sanger sequencing of EML4::ALK cDNA from Ba/F3 K1150dup cells showing a lysine insertion at residue 1150. **B** Inhibitory activity of lorlatinib and neladalkib in the indicated EML4::ALK-expressing Ba/F3 cells. Cells were treated with inhibitors for 72 h, and cell viability was analyzed using the CellTiter-Glo assay. *n* = 3 independent samples examined in three independent experiments and representative data are presented as mean ± SD. **C** ALK phosphorylation in the indicated EML4::ALK-expressing Ba/F3 cells treated with 100 nM lorlatinib or neladalkib for 4 h. **D** Phosphorylation of ALK and downstream kinases in Ba/F3 cells expressing WT or K1150dup EML4::ALK after a 4-h treatment with the indicated concentrations of lorlatinib or neladalkib. Ba/F3 models were generated using the EML4::ALK variant 3, except for the G1202R + L1196M mutant, which was expressed on an EML4::ALK variant 1 background.

To investigate whether the ALK K1150dup mutation confers resistance to the ALK-TKI used in this patient, we established Ba/F3 cells expressing EML4::ALK with the K1150dup mutation (Fig. 2A). We first assessed their sensitivity to lorlatinib using cell viability assays. For comparison, we included several clinically relevant ALK variants: the G1202R mutation, which is partially sensitive to lorlatinib, and the lorlatinib-resistant compound mutations G1202R + T1151M, G1202R + L1196M, and D1203N + L1196M^13,18^. Notably, while most mutants were generated on the EML4::ALK variant 3 background, the G1202R + L1196M mutant was expressed on an EML4::ALK variant 1 background, due to the availability of a previously established cell model. The ALK K1150dup-mutant cells showed marked resistance to lorlatinib, comparable to the lorlatinib-resistant compound mutations (Fig. 2B). Furthermore, the ALK K1150dup-mutant cells exhibited marked resistance to the next-generation inhibitor neladalkib. We treated all mutant cells with lorlatinib and neladalkib and assessed ALK phosphorylation by western blot (Fig. 2C). Consistent with the cell-viability assay results, lorlatinib failed to suppress ALK phosphorylation in all mutant cells, and neladalkib was ineffective in K1150dup and D1203N + L1196M mutants. We further assessed ALK phosphorylation and downstream signaling at increasing concentrations of lorlatinib and neladalkib (Fig. 2D). In contrast to ALK wild-type (WT) cells, both ALK phosphorylation and downstream signaling in ALK K1150dup-mutant cells remained largely active and were inhibited only at high concentrations of the inhibitors (≥300 nM). Taken together, these results demonstrate that the ALK K1150dup mutation confers resistance to lorlatinib and neladalkib.

### Gilteritinib overcomes lorlatinib resistance conferred by the ALK K1150dup mutation

**Fig. 3:**
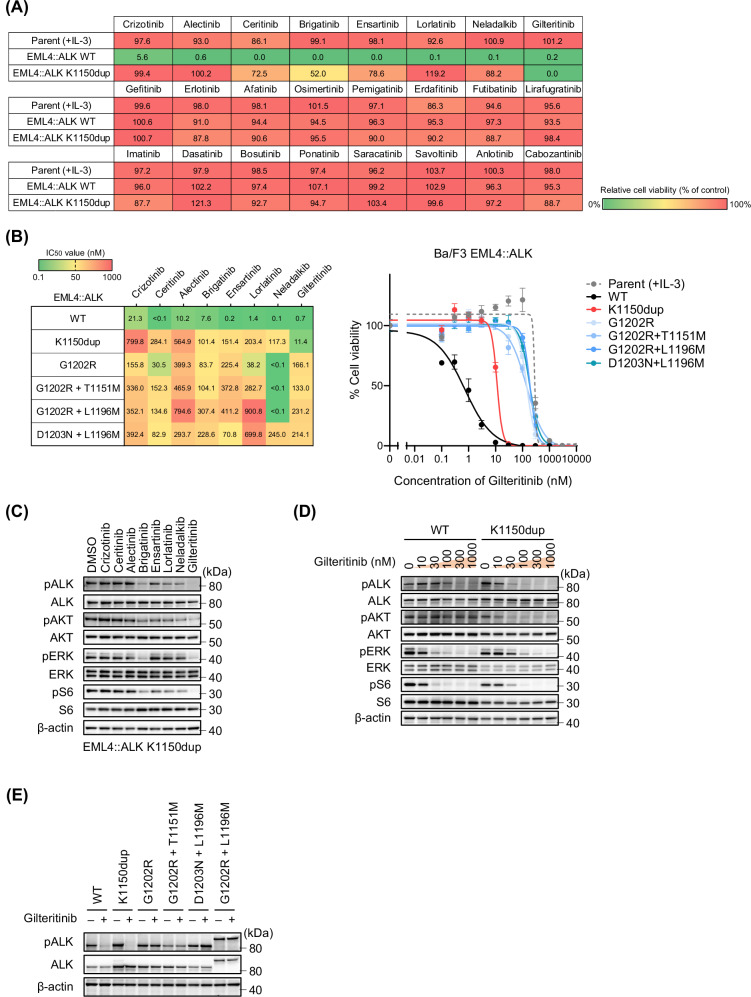
Efficacy of gilteritinib against ALK K1150dup mutation. **A** Relative cell viability of parental Ba/F3 cells (with IL-3), EML4::ALK WT, and EML4::ALK K1150dup-expressing Ba/F3 cells treated with 50 nM of the indicated inhibitors for 72 h. Cell viability was calculated relative to that of dimethyl sulfoxide-treated Ba/F3 cells and is presented as the means of at least three independent experiments. **B** Left, Graphical representation of IC₅₀ values. The indicated EML4::ALK-expressing Ba/F3 cells were treated with inhibitors for 72 h, and cell viability was assessed using the CellTiter-Glo assay. IC₅₀ values (nM) are presented as the means of three independent experiments. Right, Inhibitory activity of gilteritinib in the indicated EML4::ALK-expressing Ba/F3 cells. *n* = *3* independent samples examined in three independent experiments and representative data are presented as mean ± SD. **C** Phosphorylation of ALK and downstream kinases in Ba/F3 cells expressing EML4::ALK K1150dup after a 6-h treatment with 50 nM of the indicated inhibitors. **D** Phosphorylation of ALK and downstream kinases in Ba/F3 cells expressing EML4::ALK K1150dup after a 4-h treatment with the indicated concentrations of gilteritinib. **E** ALK phosphorylation in the indicated EML4::ALK-expressing Ba/F3 cells treated with 100 nM gilteritinib for 6 h.

Next, we sought to identify drugs that could overcome the resistance driven by the ALK K1150dup mutation. A previous study reported that the multi-kinase inhibitor gilteritinib overcomes lorlatinib resistance in ALK-rearranged cancers harboring several compound mutations^19^, and two phase 1 trials enrolling patients with stage IV ALK-positive NSCLC are currently ongoing (NCT06225427 and NCT07140016). We therefore evaluated the efficacy of gilteritinib in our Ba/F3 models. We tested a focused panel of TKIs on Ba/F3 cells expressing the ALK K1150dup mutation. At a concentration of 50 nM, only gilteritinib strongly inhibited the proliferation of ALK K1150dup-mutant cells (Fig. 3A). We also determined the IC_50_ values of seven ALK-TKIs and gilteritinib in Ba/F3 cells expressing the K1150dup mutation or other ALK mutations (Fig. 3B). Gilteritinib showed the most potent activity against the ALK K1150dup mutation among the drugs tested, exhibiting an IC_50_ of 11.4 nM. Western blot analysis further showed that only gilteritinib effectively suppressed ALK phosphorylation in K1150dup-mutant cells (Fig. 3C). ALK phosphorylation and downstream signaling in K1150dup-mutant cells were effectively suppressed at relatively low concentrations of gilteritinib (Fig. 3D). We also treated all mutant cells with gilteritinib and assessed ALK phosphorylation (Fig. 3E). Consistent with the IC_50_ results, gilteritinib selectively inhibited ALK phosphorylation in K1150dup-mutant cells. These results demonstrate that gilteritinib effectively overcomes lorlatinib resistance conferred by the ALK K1150dup mutation.

### Effect of ALK K1150dup on lorlatinib and gilteritinib binding modes and protein–inhibitor interaction frequencies

**Fig. 4:**
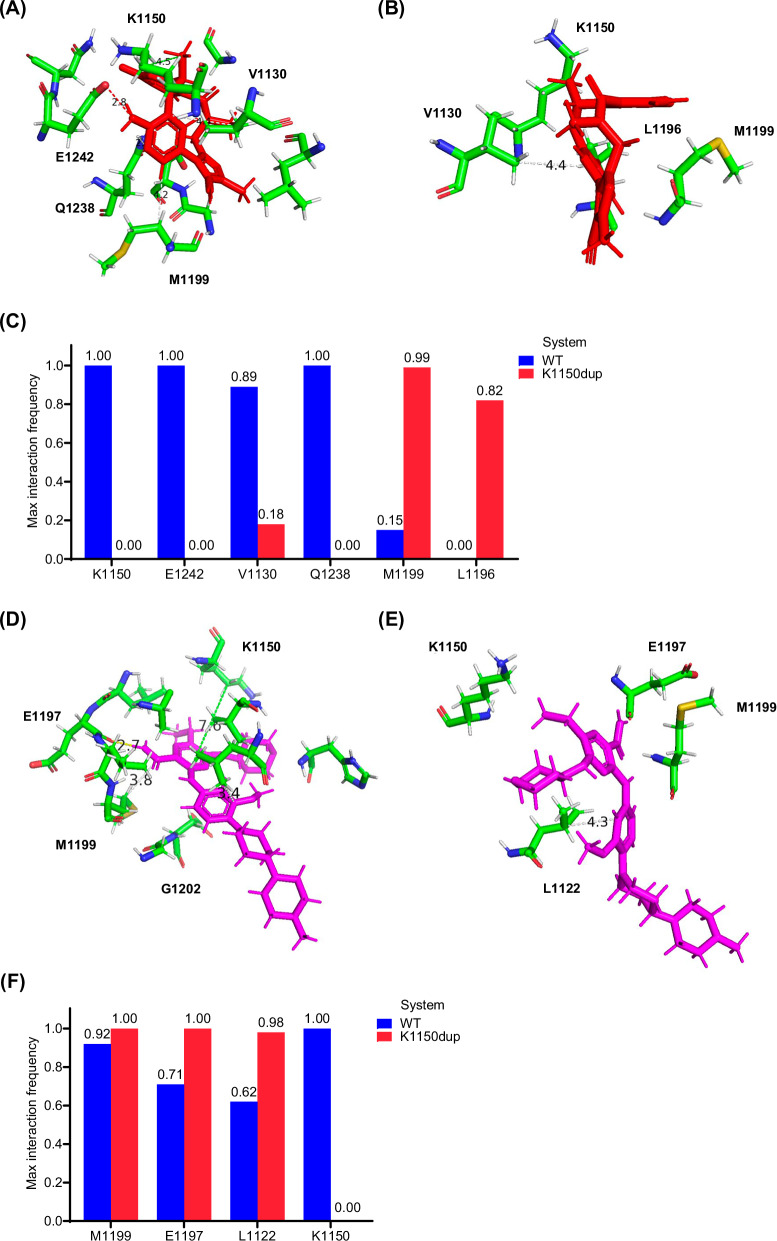
Structural modeling and predicted drug binding affinities for the ALK K1150dup mutation. **A** Experimentally derived pose of lorlatinib (red) in the binding site of ALK WT (PDB:7R7R). **B** Predicted binding pose of lorlatinib (red) in the binding site of ALK K1150dup. **C** Bar plot of interaction frequencies for lorlatinib in ALK WT and K1150dup. **D**, **E** Predicted binding pose of gilteritinib (pink) in the binding pockets of ALK WT and K1150dup. **F** Bar plot of interaction frequencies for gilteritinib in ALK WT and ALK K1150dup.

We then assessed the molecular mechanism for the differential activity of lorlatinib and gilteritinib against ALK K1150dup, by evaluating their binding modes and interactions with the ALK kinase domain using computational approaches. In the WT complex (Fig. 4A), lorlatinib is tightly anchored in the active site between E1242, Q1238, V1130 and K1150. M1199 and L1196 contribute only marginally in the ALK WT, indicating a binding mode primarily supported by the E1242–Q1238–K1150 triad together with hydrophobic packing against V1130. In the ALK K1150dup variant (Fig. 4B), modeling indicates that the insertion of an additional lysine at position 1150 perturbs this network and displaces lorlatinib toward the M1199/L1196 region, consistent with the increased ligand root mean square deviation (RMSD) in heavy atom positions and loss of stabilizing contacts reported in Table 1. Polar interactions with E1242 and Q1238 are disrupted, contacts to V1130 are reduced, and interaction with K1150 is essentially lost. The duplicated lysine side chain is oriented towards the solvent and no longer adopts a geometry compatible with stable binding, while new contacts emerge with L1196 and M1199. Accordingly, the bar plot in Fig. 4C shows a marked decrease in the interactions with K1150, E1242, Q1238 and V1130, and a concomitant increase in interactions for M1199 and L1196. Overall, ALK K1150dup replaces a well-defined polar anchoring network with a lateral binding mode dominated by hydrophobic contacts with M1199 and L1196, consistent with reduced protein-ligand complementarity and a putative reduction in lorlatinib binding.

**Table 1:** Structural deviation of ALK inhibitor binding poses

Compound	RMSD vs experimental structure (Å)	Likely impact on binding
Gilteritinib^a^	0.5	Neutral
Neladalkib	2.3	Negative
Lorlatinib	1.9	Negative
Ensartinib^a^	2.0	Negative
Brigatinib	2.3	Negative
Alectinib	1.8	Negative
Ceritinib	1.4	Negative
Crizotinib	1.5	Negative

RMSD values relative to experimental ALK structures and their predicted impact on binding.

^a^Indicates computationally generated binding poses for ALK WT.

As no experimental structure is available for the ALK:gilteritinib, we created a model of the wild-type ALK:gilteritinib using the available co-crystal structures of gilteritinib with MerTK and FLT3. The three tyrosine kinase domains of ALK, MerTK and FLT3 all share ~30–32% identity, with an observed RMSD variation in experimentally observed gilteritinib binding poses between FLT3 and MerTK of 1.3 Å. The model of ALK K1150dup displayed an RMSD of 0.5 Å to the FLT3-derived template, supporting the plausibility of the proposed gilteritinib binding mode, although this model is not derived from an experimental ALK:gilteritinib structure. In the computationally generated WT complex (Fig. 4D), gilteritinib adopts an extended pose organized around a core formed by E1197, M1199 and the G1202 region. K1150 is oriented toward the ligand and provides an additional anchor, as reflected by its high interaction frequency in Fig. 4F. In the ALK K1150dup variant (Fig. 4E), gilteritinib undergoes a moderate conformational change, while preserving its principal interaction network with E1197 and M1199, in line with the minimal changes in RMSD reported in Table 1. An additional contact with L1122 provides extra hydrophobic stabilization, reflected by increased interaction frequencies in Fig. 4F. In contrast, the contribution of K1150 falls to essentially zero in the mutant.

Together, these findings indicate a higher susceptibility of lorlatinib to ALK K1150dup-mediated resistance, whereas gilteritinib retains a more robust protein–inhibitor complementarity.

## Discussion

The clinical management of ALK-positive NSCLC has recently evolved, moving lorlatinib from a later-line treatment option to a frontline therapy of choice. The median PFS unreached at five years of follow-up in the CROWN study suggests that some patients with advanced ALK-driven NSCLC may be cured with lorlatinib^17^. Nonetheless, managing acquired resistance to lorlatinib remains a clinical challenge. The last-generation inhibitor neladalkib can overcome *ALK* compound mutations that drive lorlatinib resistance^15^. However, preclinical data indicate that lorlatinib retains activity against virtually all known single ALK mutations^11,13^, and that ALK compound resistance mutations arise under lorlatinib only when the drug is administered after a prior ALK-TKI^13^. Indeed, preliminary exposure to first- or second-generation TKIs can enable the emergence of an initial single ALK mutation, upon which additional molecular events may build to generate lorlatinib-resistant compound mutations. Given lorlatinib’s optimal on-target activity, ALK resistance mutations were therefore expected to play, at most, a limited role in resistance when lorlatinib is used as a frontline ALK-TKI.

Our findings demonstrate that on-target ALK resistance can emerge under first-line lorlatinib, contradicting current assumptions derived from clinical trials^17^. In this study, we characterized the ALK K1150dup mutation identified in a patient with ALK-positive NSCLC progressing on first-line lorlatinib. This is the first report of a mutation occurring at position K1150 in the ALK kinase domain, which has not previously been described as a mutational hotspot at progression on early-generation ALK inhibitors or on lorlatinib when administered in a sequential strategy. The study of this resistance setting in large cohorts is required to appreciate whether on-target ALK resistance in general, and ALK K1150dup in particular, represent recurrent mechanisms of resistance. Using Ba/F3 cell models, we demonstrated that the ALK K1150dup mutation confers resistance to lorlatinib and all the other ALK-TKIs including neladalkib. Consistent with these findings, structural modeling suggested that the K1150dup mutation may impair the binding affinity of lorlatinib. Notably, we found that gilteritinib, a multi-kinase inhibitor approved for FLT3-mutated acute myeloid leukemia, effectively suppresses the proliferation of ALK K1150dup-mutant cells. In contrast, other clinically approved ALK-TKIs were largely ineffective in these models. The efficacy of gilteritinib against this mutation may result from a binding mode on ALK that is distinct from lorlatinib. Because no experimental ALK:gilteritinib co-crystal structure is available, the ALK:gilteritinib complex was inferred from homologous kinase templates with modest sequence identity to ALK. Accordingly, the structural modeling and docking analyses were used to generate qualitative mechanistic hypotheses regarding inhibitor binding and the effect of K1150dup, rather than quantitative predictions of binding affinity. These results are consistent with previous reports showing that gilteritinib retains activity against certain lorlatinib-resistant compound mutations^19^. Collectively, our findings suggest that gilteritinib represents a potential therapeutic option for patients harboring the ALK K1150dup resistance mutation.

In conclusion, our study identifies the ALK K1150dup mutation as a novel mechanism of resistance to lorlatinib and suggests gilteritinib as a potential therapy for patients harboring this mutation. Our study underscores the value of systematically characterizing resistance mechanisms in precision oncology, as it reveals new treatment opportunities based on tumor molecular evolution.

## Methods

### Molecular analyses

The patient was enrolled in UNLOCK (patient ID: ST2350), an institutional program aiming to decipher mechanisms of action and resistance to innovative drugs. The tissue biopsy at the first progression to lorlatinib was analyzed with a targeted next-generation sequencing panel (ArcherDx CTL fusionplex). The tissue biopsies at the end of lorlatinib treatment were analyzed with whole-exome sequencing and bulk RNA sequencing. ctDNA was analyzed with FoundationOne Liquid CDx panel. The patient was included in the institutional clinical-molecular study STING (NCT04932525). The patient was fully informed and signed a written informed consent. This protocol was approved by the CPP (“Comité de protection des personnes”) and by ethics committees in France (French National Agency for Medicines and Health Products Safety - ANSM) and adhered to the principles in the Guideline for Good Clinical Practice and the Declaration of Helsinki.

### Cell lines

Ba/F3 cells were purchased from DSMZ and cultured in Dulbecco’s modified Eagle’s medium supplemented with 10% fetal bovine serum in the presence of IL-3 (0.5 ng/mL). Ba/F3 cells were infected with lentiviral constructs as previously reported^20^, to express the EML4::ALK variant 3 fusion with or without ALK kinase domain mutations. Ba/F3 cells harboring the EML4::ALK fusion were selected in the presence of blasticidin (21 μg/mL) and IL-3 (0.5 ng/mL) until recovery, and a second selection by culturing the cells in the absence of IL-3. EML4::ALK rearrangement and ALK kinase domain mutations were confirmed on the established cell lines by Sanger sequencing. EML4::ALK variant 1 G1202R + L1196M-expressing Ba/F3 cells were established in Japanese Foundation for Cancer Research (Tokyo, Japan).

### Site-directed mutagenesis

Lentiviral vectors expressing the EML4::ALK variant 3 were created using the pLenti6/V5 directional TOPO Cloning Kit (K495510, Thermo Fisher Scientific) according to the manufacturer’s instructions. Point mutations and in-frame insertions were introduced using the QuikChange XL Site-Directed Mutagenesis Kit (200516, Agilent) as described previously^18^. The K1150dup mutant was generated in this study using the following primers:

K1150dup Forward: CCCCTGCAAGTGGCTGTGAAGAAGACGCTGCCTGAAGTGTGCTC

K1150dup Reverse: GAGCACACTTCAGGCAGCGTCTTCTTCACAGCCACTTGCAGGGG

### Reagents

Crizotinib (S1068), Alectinib (S2762), Ceritinib (S7083), Brigatinib (S8229), Ensartinib (S2934), Lorlatinib (S7536), NVL-655 (E1961), Gilteritinib (S7754), Gefitinib (S1025), Erlotinib (S7786), Afatinib (S1011), Osimertinib (S7297), Pemigatinib (S0088), Erdafitinib (S8401), Futibatinib (S8848), Lirafugratinib (E1431), Imatinib (S2475), Dasatinib (S1021), Bosutinib (S1014), Ponatinib (S1490), Saracatinib (S1006), Savoltinib (S7674), Anlotinib (S8726) and Cabozantinib (S1119) were purchased from Selleck Chemicals.

### Cell viability assay

Ba/F3 cells were seeded at 2000 cells/well into 96-well plates in triplicate and cultured in medium containing serially diluted drugs for 72 h. Cells were subsequently incubated with CellTiter-Glo reagent (G7570, Promega), and luminescence was measured. To analyze the data, GraphPad Prism version 10.3.0 (GraphPad software) was used. IC_50_ values were determined using a nonlinear regression model with a sigmoidal dose-response in GraphPad.

### Antibodies and immunoblotting

Cells were lysed in RIPA Extraction & Lysis Buffer (89901, Thermo Fisher Scientific) supplemented with Protease and Phosphatase Inhibitor Cocktail (78444, Thermo Fisher Scientific), and boiled at 95 °C for 5 min. Protein concentrations were determined using the BCA protein assay kit (23225, Thermo Fisher Scientific). Equal amounts of protein were separated by SDS-PAGE and transferred to nitrocellulose membranes. The following antibodies were used: phospho-ALK (Y1282/1283; #9647, Cell Signaling Technology), ALK (#3633, Cell Signaling Technology), phospho-AKT (#4060, Cell Signaling Technology), AKT (#4961, Cell Signaling Technology), phospho-p42/44 ERK/MAPK (#9101, Cell Signaling Technology), p42/44 ERK/MAPK (#9102, Cell Signaling Technology), phospho-S6 ribosomal protein (S235/236; #4858, Cell Signaling Technology), S6 ribosomal protein (#2217, Cell Signaling Technology), β-actin (#PA1-183, Thermo Fisher Scientific).

### Structural modeling

Homology models were generated in PyMod3 (PyMOL plugin)^21^ using MODELLER^22^, via the standard AutoModel workflow with default optimization/refinement settings, unless otherwise specified. Docking was performed against templates of either the wild-type ALK:crizotinib (PDB:2XP2^23^) structure or the K1150dup model (created using MODELLER) using smina^24^, a fork of AutoDock vina^25^. While the experimentally available ALK:lorlatinib structure could be used as a reference (PDB:7R7R^14^) for docking, no experimentally derived template is available for an ALK:gilteritinib complex. As a result, the available complex structures of gilteritinib with FLT3 (PDB:6JQR^26^, 32% identity to ALK) and MerTK (PDB:7AB1^27^; 33% identity to ALK) were overlaid onto the wild-type ALK structure to provide a predicted reference pose for the ALK:gilteritinib complex. The RMSD on non-hydrogen atoms after prealignment between these two experimental structures (identity 31%) was 1.3 Å (no prealignment 1.9 Å). The template pose derived from FLT3 was selected as a reference based on sequence conservation. The calculated gilteritinib poses were then ranked based on their similarity (RMSD) with the template structures and the top-ranking poses used in subsequent analysis^22^. As no experimental ALK:gilteritinib co-crystal structure is available, and the complex was inferred from homologous kinase templates with modest sequence identity to ALK, the structural modeling and docking analyses are used to generate qualitative mechanistic hypotheses regarding inhibitor binding and the effect of K1150dup, rather than quantitative predictions of binding affinity.

## Supplementary information

Supplementary information

## Data Availability

WES/RNA-seq raw data files from this study are deposited at the European Genome–phenome Archive (EGA) using the accession code EGAD50000002553 and EGAD50000002554. Access to this shared dataset is controlled by the institutional Data Access Committee, and requests for access can be sent to the corresponding author. Further information about EGA can be found at https://ega-archive.org/. Any additional information required to reanalyze the data reported in this article is available upon request from the corresponding author.

## Code availability

Code availability is not applicable to this study.
