# Supplementary material for: The novel ALK K1150dup mutation mediates resistance to frontline lorlatinib and retains sensitivity to gilteritinib
Source: NPJ Precis Oncol. 2026 May 13;10:286. doi: 10.1038/s41698-026-01477-z (PMC13396518; doi:10.1038/s41698-026-01477-z)
Supplement: Supplementary file 1 — Supplementary information [file 41698_2026_1477_MOESM1_ESM.pptx]

## Slide 1
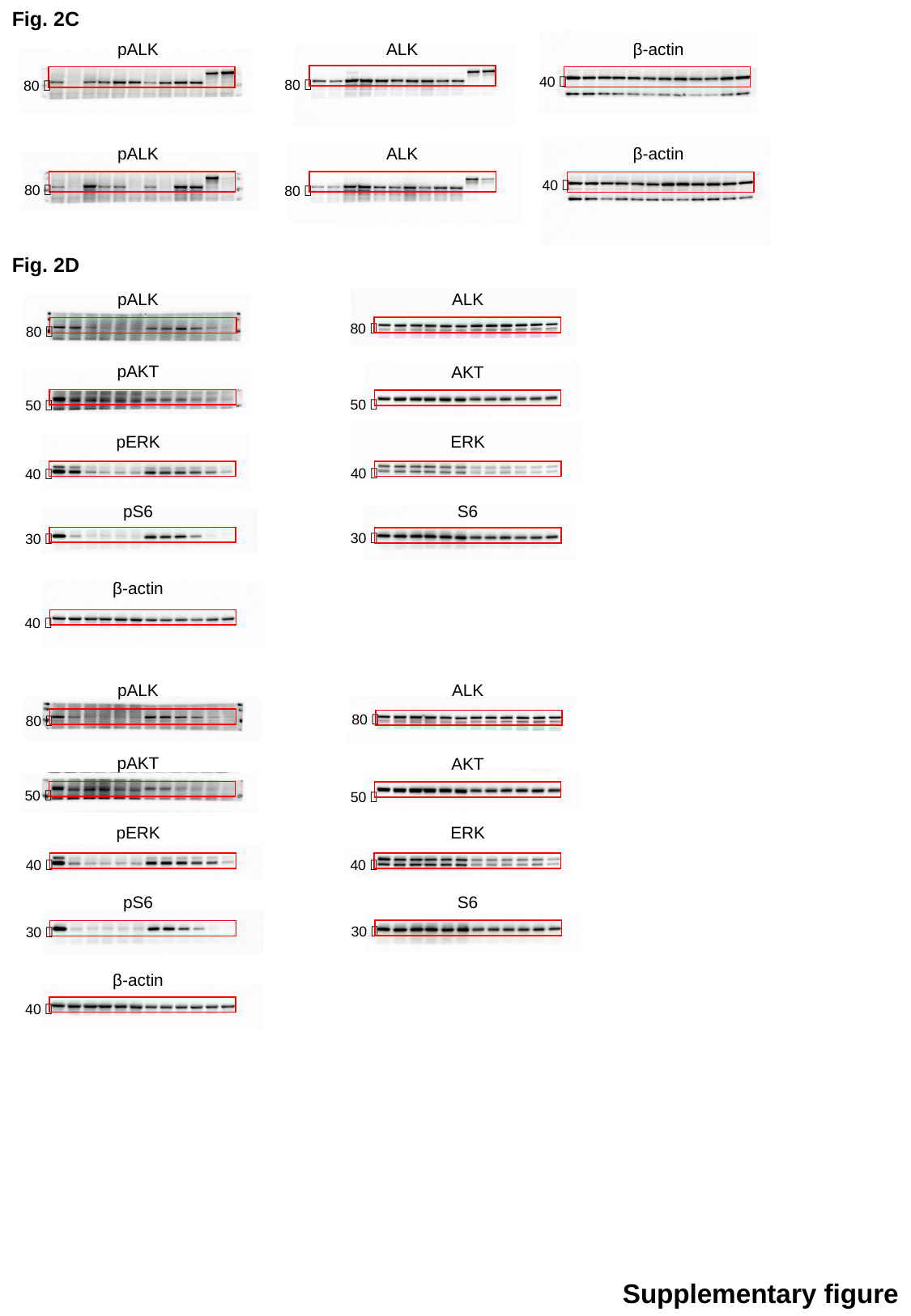

Fig. 2C
40－
pALK
ALK
β-actin
80－
80－
pALK
ALK
β-actin
40－
80－
80－
Fig. 2D
pALK
ALK
80－
80－
pAKT
AKT
50－
50－
40－
pERK
ERK
40－
pS6
S6
30－
30－
β-actin
40－
pALK
ALK
80－
80－
pAKT
AKT
50－
50－
pERK
ERK
40－
40－
pS6
S6
30－
30－
β-actin
40－
Supplementary figure

## Slide 2
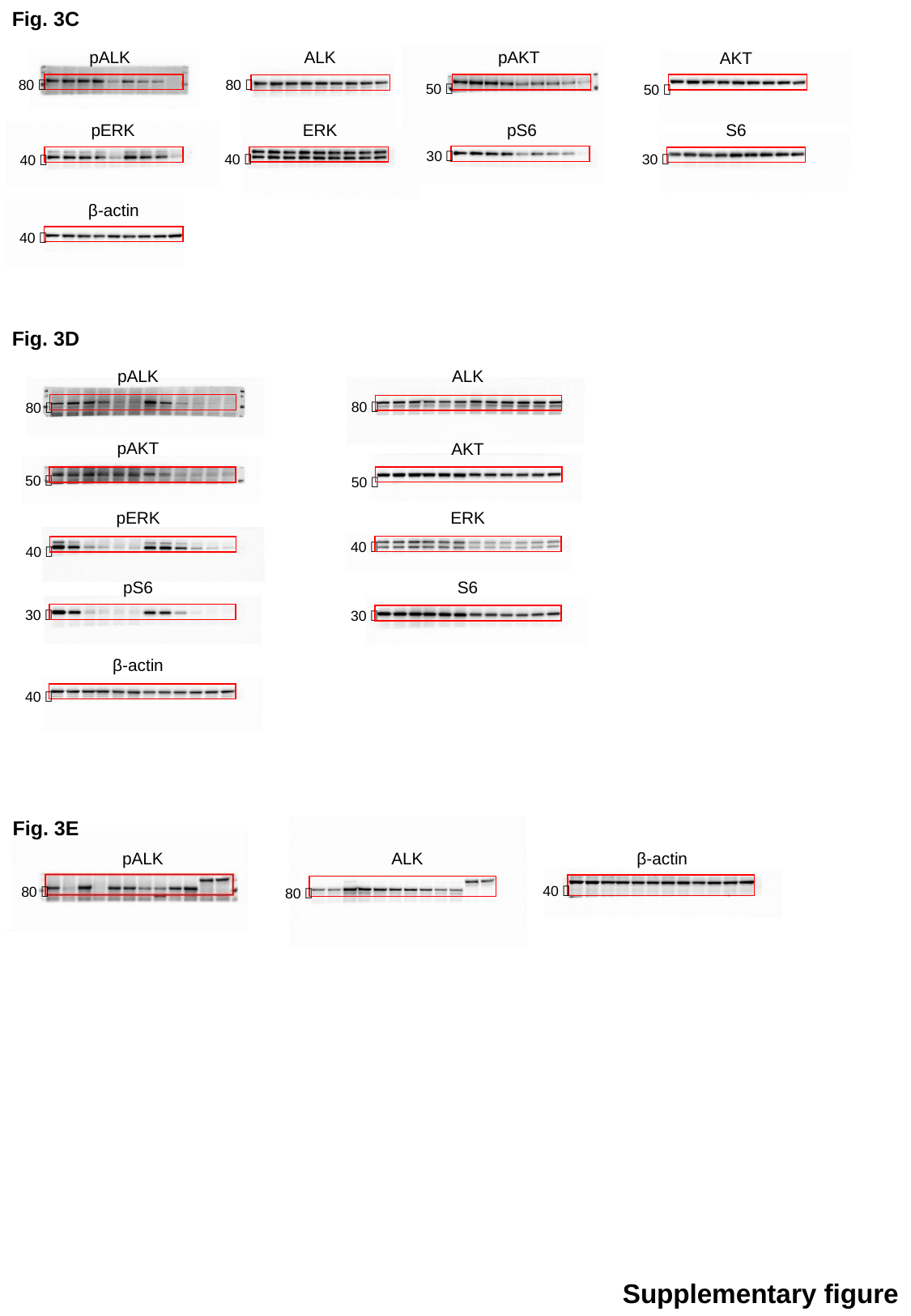

Fig. 3C
pALK
ALK
pAKT
AKT
50－
50－
80－
80－
pERK
ERK
pS6
S6
30－
30－
40－
40－
β-actin
40－
Fig. 3D
pALK
ALK
80－
80－
pAKT
AKT
50－
50－
pERK
ERK
40－
40－
pS6
S6
30－
30－
β-actin
40－
Fig. 3E
80－
pALK
ALK
β-actin
40－
80－
Supplementary figure
